# Maladaptive Liver Regeneration in Chronic Liver Disease: Cellular Mechanisms Linking Ductular Reaction, Parenchymal Extinction, and Extracellular Matrix Remodelling

**DOI:** 10.3390/jpm16100526

**Published:** 2026-10-11

**Authors:** Martyna Szymonik, Nadzieja Krasucka, Milena Krupa, Dominika Małek, Julia Płaza-Kościańczuk, Jakub Kleinrok, Weronika Pająk, Kamil Rusztyn, Maciej Mazur, Agnieszka Korolczuk

**Affiliations:** 1 Chair and Department of Clinical Pathomorphology, Medical University of Lublin, Jaczewskiego 8b, 20-090 Lublin, Poland; 64555@umlub.edu.pl (M.S.); 61207@umlub.edu.pl (N.K.); 63123@umlub.edu.pl (M.K.); 63174@umlub.edu.pl (D.M.); 63269@umlub.edu.pl (J.P.-K.);; 2 Faculty of Chemistry, University of Warsaw, Ludwika Pasteura 1, 02-093 Warsaw, Poland; kamil.rusztyn12@gmail.com (K.R.);; 3 Department of Oncology, Medical University of Warsaw, 02-091 Warsaw, Poland

**Keywords:** chronic liver disease, maladaptive regeneration, ductular reaction, extracellular matrix remodelling, parenchymal extinction, liver fibrosis, hepatocellular carcinoma

## Abstract

Chronic liver disease is characterised not only by persistent injury and fibrosis, but also by a progressive shift from adaptive hepatocyte-driven repair toward maladaptive tissue remodelling. Although liver regeneration is often discussed as a compensatory response, chronic injury may render this process structurally and functionally dysregulated, thereby promoting architectural distortion, fibrosis progression, and a microenvironment permissive for hepatocarcinogenesis. In this narrative review, we propose that this transition can be interpreted through three interconnected, histopathology-anchored endpoints: ductular reaction, extracellular matrix remodelling, and parenchymal extinction. We discuss ductular reaction as a progenitor niche phenotype that emerges when effective hepatocyte regeneration is impaired and becomes increasingly linked to portal inflammation, fibrogenic signalling, and altered cell–matrix interactions. We then examine extracellular matrix remodelling as a central tissue-level process involving activated stellate cells, portal fibroblasts, macrophages, cholangiocytes, and dysfunctional endothelial cells, resulting in collagen accumulation, increased stiffness, fibrous septa formation, and progressive reorganization of the hepatic microenvironment. Finally, we address parenchymal extinction as a morphological endpoint of often-irreversible or incompletely reversible hepatocyte loss accompanied by microvascular injury, ischaemia, and fibrotic replacement, contributing directly to architectural collapse and disease advancement. Rather than representing isolated phenomena, these three processes form a biologically integrated remodelling axis that links failed regeneration with fibrosis progression and malignant transformation. By framing chronic liver disease through these assessable histologic endpoints, this review aims to provide a more coherent pathology-oriented model of progression and to highlight mechanisms that may improve tissue-based risk stratification and future translational research.

## 1. Introduction

### 1.1. Chronic Liver Disease—Definition and Clinical Significance

Chronic liver disease (CLD) is a progressive inflammatory and fibrotic process lasting more than six months. It leads to fibrosis and cirrhosis. Cirrhosis is the final stage of CLD; it disrupts the architecture of the liver, causing nodule formation, vascular reorganisation, neoangiogenesis and extracellular matrix (ECM) deposition [1]. Liver fibrosis, and in some cases even cirrhosis, can be reversed following successful treatment of the underlying disease. Following liver injury, a healing process ensues, involving: increased production and deposition of ECM, inflammation, and myofibroblast proliferation. Biopsy remains the gold standard for diagnosing liver fibrosis. Biopsy is useful for monitoring disease progression and planning treatment; however, due to its limitations, non-invasive tests—blood tests and radiological methods—have been developed. Commonly used indirect markers include the FIB-4 score and the AST-to-platelet ratio (APRI). Direct markers include the Enhanced Liver Fibrosis Score (ELF), Hepascore and Fibrospect II [2]. The prevalence of CLD is steadily increasing, particularly in developing countries. The prevalence, incidence and mortality rates for CLD were higher among men, and the incidence rate increased most markedly among those aged 15 to 49 years [3].

### 1.2. Fibrosis and the ECM

Active hepatic stellate cells (HSCs) play a key role in the development of liver fibrosis. In cases of chronic damage, they are responsible for the excessive production of collagen and other ECM components, leading to progressive fibrosis. Excess ECM is a marker of CLD, and its accumulation and alteration are significant stages in disease progression [4].

The extracellular matrix (ECM) is a complex network of macromolecular proteins that provide structural support to tissues and are involved in the development and maintenance of tissue homeostasis. The interaction between cells and the ECM is bidirectional: cells receive signals from the matrix and subsequently remodel the ECM by replacing old proteins with new ones via integrins. ECM proteins are important mediators of the loss of HSC function during the progression of liver fibrosis, the dysregulation of which is a common feature of all fibrotic diseases. Proteins comprising the ECM include: collagen, elastin, fibronectin and laminins. A shift in the homeostasis between ECM proteins and their specific partners leads to inflammation and the progression of liver fibrosis, which results in increased morbidity and mortality [5].

### 1.3. Adaptive Regeneration

Adaptive regeneration is the liver’s physiological response to acute damage and serves to restore its normal structure and functions. The liver possesses an extraordinary capacity for regeneration, which stems from the high proliferative potential of hepatocytes. The regeneration process is compensatory in nature and involves the proliferation of the remaining, normal hepatocytes following partial loss of parenchyma.

Adaptive regeneration is the liver’s physiological response to acute damage and serves to restore the organ’s tissue function and mass. The liver possesses a regenerative capacity in which the proliferation of surviving hepatocytes plays a significant role. Following acute liver injury or partial loss of parenchyma, hepatocyte proliferation is activated as part of a regulated and coordinated process that enables the restoration of lost parenchymal mass and the maintenance of liver function. Prolonged or chronic damage may disrupt this coordinated regenerative response and promote abnormal repair, characterised by impaired regeneration and progressive structural changes, including fibrosis [6].

### 1.4. Maladaptive Regeneration

In cases of chronic liver damage, the body’s physiological repair mechanisms fail (failure of adaptive repair), leading to the development of maladaptive regeneration.

Maladaptive regeneration is a condition in which the liver’s normal repair response becomes permanently activated and dysregulated as a result of chronic damage. Instead of leading to parenchymal repair, this response becomes chronic, disorganised and associated with fibrosis, remodelling of the liver’s architecture, and impairment of its function and microcirculation. During chronic liver damage, the mechanisms responsible for liver regeneration remain in a state of constant activation. This process leads to excessive production of ECM by activated hepatic stellate cells. This results in the deposition of fibrous tissue and pathological remodelling of the liver’s architecture. This process is regulated by developmental pathways that are physiologically responsible for the reconstruction of the liver parenchyma, such as: Notch, Hedgehog, Hippo-YAP-TAZ (Yes-associated protein-transcriptional coactivator with PDZ-binding motif) and WNT-beta-catenin. Fibrosis is part of pathological regeneration rather than a separate process. In contrast, in cases of acute liver injury, effective regeneration occurs through the proliferation and hypertrophy of the remaining hepatocytes, leading to the restoration of normal liver structure and function. This process is reflected in three histopathologically interrelated manifestations, namely ductular reaction, ECM remodelling, and parenchymal extinction, which are considered endpoints of maladaptive regeneration. These endpoints should not be interpreted as obligatorily sequential or mutually exclusive; rather, they may coexist and reinforce one another to different degrees depending on the aetiology, stage, and vascular context of chronic liver injury. Chronic inflammation and associated microvascular thrombosis induce episodes of ischemia, directly driving the process of PE and the collapse of lobular structures. The depletion of functional hepatocytes triggers a compensatory response in the form of DR, forming a biliary reparative complex. Within this microenvironment, senescent reactive ductular cells (RDCs) secrete angiogenic factors (e.g., VEGF) and profibrogenic mediators (SASP), stimulating myofibroblasts to pathologically remodel the extracellular matrix (ECM). Consequently, the pathologically stiffened ECM and associated sinusoidal capillarization form a mechanical barrier that disrupts metabolic exchange and deepens tissue hypoxia, thereby propagating subsequent cycles of parenchymal extinction [7,8].

### 1.5. Histopathological Endpoints

#### 1.5.1. Ductular Reaction

One of the earliest and most readily identifiable histopathological signs of maladaptive regeneration is the ductular reaction. The ductular reaction (DR) is a complex cellular response occurring during chronic liver damage. It involves the proliferation of cholangiocytes and hepatocytes, as well as progenitor cells in the liver, which participate in ECM remodelling processes. The severity of the DR correlates positively with the severity of liver fibrosis. As the severity of fibrosis increases, increased expression of markers such as EpCAM/KRT7 is observed. KRT7 is used to assess expression in biopsies from patients with primary biliary cholangitis (PBC), chronic hepatitis B (CHB) and non-alcoholic fatty liver disease (NAFLD). Progenitor cells and reactive cholangiocytes actively participate in fibrogenesis [7]. DR is a histological marker, whilst ECM activation, remodelling and fibrogenesis lead to changes in liver architecture [8].

From a practical histopathological perspective, the ductular reaction should be assessed in haematoxylin and eosin (H&E)-stained sections, taking into account the number, morphology, complexity, and distribution of the ductal structures. Key morphological features include: an increased number of fine ductal-like structures, often lined by cuboidal epithelium, accompanied by inflammatory cells and fibrosis of varying severity around the portal spaces and their vicinity. This reaction is most pronounced in the portal spaces and periportal areas, although in more extensive forms it may involve elongated, branched or irregular ductal-like structures invading the adjacent parenchyma. Immunohistochemical staining using antibodies against CK7 and CK19 may facilitate the identification of ductal-like structures and the assessment of their distribution, particularly when the reaction is poorly visible in H&E staining. It should be borne in mind that these markers indicate a bile duct/intraductal epithelium phenotype and should not, in themselves, be interpreted as evidence of origin from progenitor cells. The differential diagnosis should consider: bile duct proliferation associated with biliary tract diseases or obstruction; reactive ductal proliferation accompanying hepatocyte damage; and glandular changes of a regenerative or neoplastic nature. The interpretation of the findings must take into account the clinical and histological context. In the case of fine-needle biopsies, the severity of the periportal reaction may be underestimated due to limitations in obtaining material from the portal spaces and periportal areas. Therefore, a qualitative or semi-quantitative approach is preferred when assessing the reaction, one which takes into account the extent, complexity and degree of spread within the parenchyma, rather than relying on a single immunohistochemical marker. Liver biopsy specimens containing portal spaces are suitable for assessing the portal tract reaction. Resection specimens or organs removed during transplantation allow for a more comprehensive assessment of the spatial distribution of this reaction [9].

#### 1.5.2. Parenchymal Extinction

Another, more advanced manifestation of maladaptive regeneration is parenchymal extinction. Parenchymal extinction is a process characterised by irreversible loss of hepatocytes, their replacement by fibrous tissue, changes in the sinusoidal structure, and obstruction of small portal and hepatic veins. Changes in microcirculation lead to progressive parenchymal loss with increasing impairment and vascular remodelling. This is accompanied by a reaction of the bile ducts and collapse of the hepatic trabeculae due to ischaemia [10]. The histopathological assessment of hepatic parenchymal necrosis is based primarily on architectural changes. In H&E preparations, a characteristic finding is the focal loss of adjacent hepatocytes with collapse or distortion of the remaining hepatic structure. The change may be associated with the convergence of portal and hepatic venous structures and with evidence of vascular or sinusoidal damage. Reticulin staining may be very useful to demonstrate disruption or collapse of the normal hepatic framework and hepatocyte lamellae. As the vascular component may be difficult to visualise in small biopsy specimens, interpretation should integrate H&E morphology with reticulin staining and where necessary with an additional assessment of the hepatic vascular system. Confluent necrosis, ischaemic damage, fibrotic scarring and regenerative atrophy may exhibit partially overlapping morphological features and should be considered in the differential diagnosis. Liver parenchymal necrosis can be assessed qualitatively or semi-quantitatively, depending on the number, extent and distribution of the lesions. Specimens obtained from resections and explants are particularly valuable as they preserve a greater amount of tissue structure; limited needle biopsies can underestimate focal parenchymal necrosis [11].

Chronic liver damage leads to the synthesis of excess extracellular matrix (ECM), fibrosis, and the sustained activation of signalling pathways involved in liver development and regeneration. During the course of CLD, there is excessive activation of developmental pathways: Notch, Hedgehog, and Hippo-YAP/TAZ. Physiologically, these pathways are responsible for maintaining appropriate levels of cell proliferation. Excessive activation of the aforementioned pathways in CLD causes fibrosis and the formation of pro-oncogenic microenvironments, enabling cells to transition to a precancerous state and leading to the development of hepatocellular carcinoma (HCC) [7]. Matrix metalloproteinases (MMPs) and tissue inhibitors of metalloproteinases (TIMPs) play important roles in ECM remodelling. MMPs are enzymes responsible for the degradation and remodelling of extracellular matrix components, whereas TIMPs are endogenous inhibitors of MMP activity. The balance between MMPs and TIMPs regulates ECM degradation, and its disruption may lead to excessive ECM accumulation and fibrosis [10].

Therefore, understanding the mechanisms of pathological liver regeneration is essential for identifying the progression of CLD and the mechanisms leading to the development of neoplastic processes.

### 1.6. Aim of the Study

The aim of this study is to present the mechanisms of extracellular matrix remodelling and their role in the progression of chronic liver disease, with particular emphasis on the processes of fibrogenesis and pathological regeneration, and to interpret the course of CLD through three interrelated endpoints: ductular reaction, ECM remodelling and parenchymal extinction.

### 1.7. Literature Search and Review Approach

This narrative review was based on a literature search conducted in the PubMed database. The search focused on publications addressing chronic liver disease, maladaptive liver regeneration, ductular reaction, extracellular matrix remodelling, liver fibrosis, parenchymal extinction, and hepatocarcinogenesis. Relevant original studies, reviews, and selected mechanistic and translational reports were considered. Additional publications were identified by screening the reference lists of relevant articles. The literature was selected according to its relevance to the histopathological and molecular mechanisms discussed in this review. Owing to the narrative character of the article, no formal systematic-review protocol, risk-of-bias assessment, or quantitative synthesis was performed.

## 2. ECM Remodelling/Fibrogenesis as an Endpoint

### 2.1. Characteristics of Fibrosis as a Chronic Injury

Liver fibrosis is a pathological process resulting from long-term liver damage, abnormal repair, and the excessive deposition of collagen fibres and ECM. Progressive fibrosis leads to liver failure, cirrhosis, hepatocellular carcinoma (HCC) and death. The most common causes of this condition are considered to be: HBV, HCV, alcoholism, MASH, cholestasis, and exposure to drugs and toxins [12].

Chronic liver damage is characterised by excessive accumulation of ECM components. During fibrogenesis, the physiological process of ECM remodelling undergoes changes, leading to excessive deposition of its components—primarily collagens—and to disturbances in its homeostasis. Signals originating from the ECM induce the activation of hepatic stellate cells, which are the primary source of the ECM and constitute the most numerous population of myofibroblasts in the liver. Furthermore, the ECM may exacerbate fibrogenesis by regulating adhesion signals and storing growth factors such as TGF-beta [5].

In this study, ECM remodelling is not merely regarded as a passive indicator of scarring, but also as a dynamic and regulated process that reflects an imbalance between fibrogenesis and fibrolysis. Changes in the composition, organisation and degradation of the ECM lead to a remodelling of the liver’s architecture and microcirculatory disturbances, which have a direct impact on the function and regenerative processes of hepatocytes. The ECM can be regarded as a histopathological endpoint of disease progression, integrating both the structural and functional aspects of maladaptive regeneration. Importantly, the amount of matrix identified in a biopsy does not necessarily reflect current fibrogenic activity, because matrix deposition, degradation, and remodelling are dynamically regulated and may persist despite removal or suppression of the injurious stimulus [10].

From a histopathological perspective, the remodelling of the extracellular matrix (ECM) should be assessed by analysing the quantity, distribution and organisation of collagen deposits, as well as the resulting changes in liver architecture. On H&E-stained sections, the presence of fibrosis may be indicated by widening of the portal or periportal spaces, perisinusoidal or pericellular fibrosis, the formation of fibrous septa, and bridging fibrosis, with advanced remodelling leading to distortion of the liver’s architecture and cirrhosis [13,14]. Masson’s trichrome staining and Sirius red staining are most commonly used to demonstrate collagen deposition, whilst reticulin staining is particularly useful for assessing the integrity of the hepatic skeleton and the organisation of hepatocyte lamellae [13]. Established semi-quantitative systems for grading liver fibrosis include the METAVIR, Ishak, NASH Clinical Research Network (NASH CRN), and Laennec systems, with the choice of scoring system depending on the underlying liver disease and the pattern of fibrosis [13,14]. Where the endpoint is the quantity of collagen itself rather than the degree of fibrosis, a quantitative assessment can be obtained through digital image analysis of Sirius red-stained sections and calculation of the collagen proportionate area (CPA).

Importantly, the degree of fibrosis determined on the basis of histological examination and the direct quantitative measurement of collagen are related but distinct measures, as the CPA method provides a continuous assessment of collagen content but does not fully reflect the information regarding tissue architecture taken into account in the conventional classification of fibrosis grades [15].

### 2.2. Composition and Remodelling of the ECM

There are two main types of extracellular matrix. The first, dominant type is the basement membrane (BM), which consists primarily of laminins, nidogen/entactin, heparan sulphate proteoglycans and type IV collagen. The second type is the interstitial matrix (IM), consisting of type I, III and IV collagen, elastin, fibronectin and tenascin [12]. One of the key enzymes involved in ECM remodelling processes is lysyl oxidase (LOX). LOX catalyses collagen cross-linking and activates the epithelial-mesenchymal transition (EMT), thereby promoting the migration and development of one of the most aggressive solid tumours, LIHC [5].

ECM stiffness is an important factor regulating HSC behaviour. Changes in the ECM affect motility, adhesion, differentiation, and skeletal reorganisation. Mechanical stimuli are detected by HSC surface receptors and integrins. An increase in matrix stiffness promotes HSC activation through cytoskeletal modulation and exacerbates inflammation and fibrogenesis in tissues [12].

### 2.3. HSCs as a Key Effector of Fibrogenesis

Activated hepatic stellate cells (HSCs) are located in the space of Disse. Under conditions of chronic injury, they exhibit a contractile, proliferative and fibrogenic phenotype. HSCs promote the recruitment and infiltration of immune cells through the production of pro-inflammatory cytokines and the expression of adhesion molecules—ICAM-1 and vascular cell adhesion molecule-1 (VCAM-1). The migration of activated HSCs to sites of injury is a factor favouring scar formation. HSCs, liver sinusoidal endothelial cells (LSECs) and hepatic macrophages form the liver microcirculatory microenvironment, which regulates vascular tone and controls inflammation [16].

Interactions between hepatocytes, HSCs, LSECs and resident immune cells—Kupffer cells—in the pathological microenvironment lead to ECM deposition, impaired parenchymal cell regeneration and gradual decompensation of liver function [17].

Following liver injury, hepatic macrophages—comprising KCs and monocyte-derived macrophages (MoMFs)—recognise a variety of stimulants—DAMPs, MOPPs, ROS and PAMPs—to produce cytokines and chemokines that amplify the inflammatory response. Furthermore, hepatic macrophages secrete TGF-beta and PDGF, which leads to the activation of HSCs, promoting their differentiation into collagen-producing myofibroblasts responsible for the excessive accumulation of ECM in the liver [12].

As CLD progresses, LSECs undergo a process of capillarisation, thereby becoming pro-inflammatory, profibrotic and prothrombotic cells; they maintain a microenvironment that prevents CLD regression. Reversal of the capillarisation process promotes regression of fibrosis and portal hypertension. Furthermore, the vasoprotective phenotype of LSECs reduces HSC activation and maintains the ECM microenvironment [18].

Intrahepatic cholangiocytes, proximal intrahepatic cholangiocytes and HSCs reciprocally alter their gene expression profiles, with increased proliferation of intrahepatic cholangiocytes and activation of HSCs via the WNT pathway. The regulation of cholangiocyte and HSC proliferation and activation occurs via GAL, histamine and leptin during cholangitis and fibrosis in MDR2-knockout mice [19].

### 2.4. Regulation of ECM Degradation—MMPs/TIMPs

Matrix metalloproteinases (MMPs) and tissue inhibitor of metalloproteinases (TIMPs) play key roles in ECM turnover and the fibrotic process. MMPs contribute to ECM degeneration, whereas TIMPs inhibit MMp activity. MMPs can degrade the ECM, which holds great potential for the treatment of liver fibrosis. MMPs are a family of calcium- and zinc-dependent proteolytic enzymes. TIMPs, on the other hand, are responsible for inhibiting MMP activity and do not damage physiological liver tissue. When MMPs and TIMPs are out of balance, the process of ECM deposition intensifies, which in turn accelerates and exacerbates liver fibrosis. As liver cell damage worsens, the secretion of TIMP-1 and TIMP-2 by HSCs increases, inhibiting MMP activity and preventing the degradation of the accumulated matrix [19].

### 2.5. The Inflammatory Microenvironment and Inflammasomes

Inflammasomes are intracellular protein complexes found in liver cells and immune system cells. They are molecular factors responsible for liver damage, but they may also lead to the identification of new diagnostic or prognostic biomarkers, as well as more effective therapies. In the early stages of liver disease, inflammasome activation serves a protective function, eliminating pathogens and limiting damage associated with PAMPs/DAMPs. Their chronic activation leads to excessive release of pro-inflammatory cytokines, intense recruitment of immune cells and prolonged activation of HSCs, which promotes the progression of inflammation and fibrosis. Inflammasome activation occurs via Toll-like receptors (TLRs) and NF-κB.

This process leads to the release of numerous inflammatory mediators. As the disease progresses, a type 2 cellular response begins to dominate in the liver, promoting tissue repair and fibrosis. Inflammasomes link acquired and innate immunity, as their activation influences the activation of CD4+ and CD8+ cells. Inflammasomes have been described in macrophages and epithelial cells, where they contribute to maintaining barrier function, as well as in liver Kupffer cells, haematopoietic stem cells, and hepatocytes during liver injury, thereby contributing to the process of hepatic fibrosis. Inflammasome biomarkers and surrogates are used in scientific research and have enabled an understanding of the role of the NLRP3 inflammasome in human liver diseases [20].

### 2.6. ROS in the Progression and Regression of Fibrosis

Reactive oxygen species (ROS) play a dual role in the process of liver fibrosis, as they mediate hepatocyte damage and inflammation whilst also modulating the resolution of fibrosis. At low concentrations, ROS are key secondary messengers in physiological oxidative stress signalling. This is a controlled, beneficial redox reaction, essential for proper cell function. An uncontrolled increase in ROS, characterised by an imbalance in the oxidative/antioxidative balance, leads to oxidative damage to lipids and DNA, ultimately causing tissue damage. ROS activate HSCs; therefore, in addition to direct cellular damage, the accumulation of ROS activates signalling pathways that exacerbate the inflammatory process and fibrosis [17].

### 2.7. Cell Therapies

Stem cells represent a promising therapeutic strategy due to their ability to self-renew over the long term and differentiate into various cell types. We distinguish between embryonic stem cells (ESCs) and adult stem cells: mesenchymal stem cells (MSCs) and haematopoietic stem cells (HSCs) [21].

Studies have shown that MSCs may potentially counteract fibrosis. ADMSCs (adipose-derived mesenchymal stem cells), when injected into rats with liver fibrosis, reduced the degree of fibrosis and collagen deposition, as well as the activation of the Hippo signalling pathway, whose primary role is to limit tissue growth and regulate proliferation, differentiation and cell migration within an organ. The Hippo signalling pathway is considered a key regulator of HSCs in acute liver injury [22]. Transplantation of bone marrow-derived stem cells (BMSCs) significantly attenuates liver fibrosis. RNA sequencing has shown that the REDD1 protein is a regulator of BMSC-based anti-fibrotic liver therapy and is up-regulated in the liver tissues of patients with PBC and in mice with liver fibrosis. Overexpression of REDD1 inhibits HSC activation by impairing their autophagy, which enhances the therapeutic efficacy of BMSCs, improves liver function, reduces liver damage and attenuates fibrosis [23].

### 2.8. Biomarkers of Progression and Regression

Histopathological examination is the gold standard for assessing the progression of liver fibrosis. Identifying reliable biomarkers of fibrosis progression and regression represents a key translational challenge. The strongest indicator of fibrosis regression among non-invasive methods is a reduction in liver stiffness assessed by elastography. However, this parameter does not replace liver biopsy. In assessing ECM turnover and predicting the progression of CLD, plasma biomarkers such as PRO-C3, TIMP-1, collagen IV and glycoprotein are evaluated. PRO-C3 reflects type III collagen formation by measuring a neo-epitope generated by the specific proteolytic cleavage of the N-terminal propeptide of type III collagen. Bile acids were lipids showing a significant reduction in patients with histological evidence of regression, which is a promising biomarker for monitoring fibrosis regression in advanced CLD. New, as yet unverified biomarkers in relation to histology include angiopoietin-2 (ANG-2) and microRNA-223-3p [7].

In patients suffering from biliary atresia, which is characterised by marked liver fibrosis, CXCR2 is activated by the action of IL-8. As a result, neutrophil extracellular traps (NETs) are formed and HSCs are activated, which increases the production of collagen and ECM. This, in turn, increases the expression of alpha-SMA and COL1A1, which are markers of fibrosis [24].

## 3. Ductular Reaction as a Repair Phenotype

Ductular reaction (DR) is a complex, heterogeneous cellular response induced by injury in both acute and chronic liver diseases and is associated with the restoration of liver homeostasis [25]. DR is commonly observed in biliary diseases, such as primary biliary cholangitis (PBC), primary sclerosing cholangitis (PSC), and biliary atresia (BA) [26]. It can also be identified in various liver conditions, including alcoholic or non-alcoholic steatohepatitis and viral hepatitis [27]. DR primarily consists of hyperproliferating or reactive cholangiocytes and hepatocytes or hepatic progenitor cells (HPCs), but this phenomenon also includes a reaction involving other liver tissues or cells, such as the stroma, inflammatory cells, and liver-infiltrating cells, including bone marrow-derived macrophages [26]. It should be highlighted that DR is not exclusively maladaptive. During the initial or acute phases of injury, it serves a crucial adaptive function by facilitating bile drainage and supporting early tissue repair through the restoration of hepatobiliary junctions [7]. However, under conditions of chronic exposure to a damaging agent when the physiological proliferative potential of hepatocytes is permanently exhausted and the damaging trigger is not eliminated, this originally reparative mechanism shifts to a maladaptive state [28]. The histopathological hallmark of this maladaptive process is the expansion of dysmorphic ductular structures beyond the portal tracts into the lobules, alongside the accumulation of reactive ductular cells (RDCs) and their transition into a state of cellular senescence. Consequently, these senescent RDCs become apoptosis-resistant and secrete a pro-inflammatory and profibrogenic profile (SASP), which continuously drives the activation of portal myofibroblasts [7,28]. In this process, repair mechanisms become integrated with progressive fibrosis and a pro-inflammatory that, instead of restoring parenchymal function, drives further disease progression [25]. In a clinical context, the extent of the DR at the time of diagnosis of PBC constitutes a significant prognostic marker; its intensity correlates with higher alkaline phosphatase levels, a poorer response to ursodeoxycholic acid treatment, and shorter estimated patient survival [29].

The collapse of the bile canalicular network constitutes an early, etiology-independent defect of hepatocyte injury that induces DR. The primary goal of DR is the restoration of functional bile secretion, which is crucial for protecting the liver parenchyma against the cytotoxic effects of bile acids [30,31]. The functional and complementary ductal systems formed during this reaction restore continuity between the injured parenchyma and the existing biliary tree [9,30]. Three-dimensional analyses have demonstrated that in chronic liver diseases of various etiologies, DR is characterised by high spatial complexity correlating with the progression of fibrosis, and it serves primarily as a system of functional hepato-biliary junctions maintaining the continuity of bile drainage, less frequently acting as a direct source of hepatocyte regeneration [32]. It should be emphasised that DR is complex and multiphasic in common bile duct occlusion models (CBDOs). It encompasses not only classical cholangiocyte proliferation but also hepatocyte transdifferentiation and progenitor cell activation. A key diagnostic aspect remains the distinction between the preproliferative, proliferative, and paraproliferative phases, which together determine the ability of the biliary bed to form collateral circulation in response to complete cholestasis [33].

This process is supported by intense neoangiogenesis. In the pathogenesis of chronic liver diseases, DR structures, by secreting the SLIT2 ligand, become a key initiator of intrahepatic angiogenesis through the activation of the ROBO1 receptor on small vessel endothelial cells. In addition to the direct stimulation of new blood vessel formation, the SLIT2 factor secreted by the DR also promotes the progression of organ fibrosis by interacting with hepatic stellate cells [34].

DR constitutes a complex histopathological phenomenon characterised by the presence of ductular profiles lined by cells with a ductular phenotype on microscopic images of the liver [26]. It is a dynamic expansion of dysmorphic structures of biliary epithelial cells and liver progenitors, which primarily localise within the portal triads and, as the injury progresses, infiltrate the liver parenchyma [28]. DR can take various morphological forms depending on the etiology of the liver disease and the degree of parenchymal damage, and on this basis, Desmet distinguished four types of this reaction (Table 1) [25].

The DR can also be classified based on the immunohistochemical profile of the cells forming the ductular structures. Analysis of the expression of selected markers, such as CD56 (NCAM), epithelial membrane antigen (EMA), and the hepatocyte marker CD10, allows for the distinction of distinct DR subtypes, which reflect varying degrees of cell differentiation and their regenerative potential. Based on these markers, three basic types of ductular reaction are distinguished: primary, differentiating, and obstructive (Table 2). Additionally, from a topographical perspective, DR is divided into “non-invasive”, “minimally invasive”, and “invasive” based on the extent of its spread within the lobular parenchyma [35].

Cytoskeletal proteins are commonly used for basic immunohistochemical identification and visualisation of DR structures, including cytokeratin 7 (CK7) and cytokeratin 19 (CK19), as they constitute the classical protein components of the mature cholangiocyte cytoskeleton [27]. It should be noted, however, that these markers do not allow for the histological differentiation of highly specialised, damage-susceptible large cholangiocytes from the plastic, stress-resistant subpopulation of small cholangiocytes, whose compensatory proliferation and subsequent transdifferentiation directly drive the development of DR in the course of chronic liver diseases. Due to the close association of DR with the activation of HPCs, the detection of early-stage differentiation markers is also of crucial importance; therefore, molecules such as the epithelial cell adhesion molecule (EpCAM) and the transcription factor SOX9 are used as HPC markers; however, they are not specific, as they are also expressed by cholangiocytes [25,26,27]. SOX9 plays a crucial role in the process of hepatocyte transdifferentiation into ductular cells, and a high number of SOX9-positive hepatocytes is a favourable prognostic factor during the Kasai procedure, which correlates with prolonged native liver survival in children with biliary atresia [36]. Under conditions of chronic liver injury, an important marker of reactive cells within DR is lipocalin 2 (LCN2), which acts as a key inflammatory mediator responsible for macrophage recruitment and the exacerbation of fibrosis in the injured organ [37].

It is worth adding that soluble cytokeratin 19 fragments (CYFRA21-1) also serve as a serological marker of DR, whose serum levels correlate with the severity of portal hypertension and mortality during advanced chronic liver disease (ACLD). This marker remains stable and independent of conventional biochemical parameters and the MELD score, allowing for more precise prediction of clinical outcomes regardless of the disease etiology [38]. Under physiological conditions, liver regeneration occurs primarily through the proliferation of mature hepatocytes [39]. In the case of chronic or severe injury, the replicative capacity of hepatocytes may be limited, leading to the activation of alternative regenerative mechanisms, including the proliferation of progenitor cells and cholangiocytes within the periportal niche. The most important factors inducing DR include chronic inflammation, cholestasis, oxidative stress, immunological, genetic, or toxic factors, and metabolic disorders (Table 3) [25,27].

The initiation and maintenance of proliferation during DR is a multilevel process, regulated by signalling cascades, interactions with the microenvironment, and metabolic mechanisms (Table 4) [26,28].

The complexity of the pathophysiological mechanisms underlying DR makes the identified signalling pathways promising therapeutic targets in the treatment of chronic liver diseases. Modulation of the organ microenvironment and direct inhibition of the pathological expansion of biliary epithelial cells can effectively prevent the progression of fibrosis to the stage of cirrhosis. However, the development and implementation of these strategies require further advanced research and the search for new drugs or the repositioning of existing substances that will target these pathways in a specific manner (Table 5).

## 4. Crosstalk Between Ductular Reaction and Extracellular Matrix Remodelling

Current pathophysiological studies indicate a strong association between the occurrence of ductular reaction (DR) and liver fibrosis, particularly in chronic liver diseases [60,61,62,63]. We distinguish the following conditions: primary sclerosing cholangitis (PSC), primary biliary cholangitis (PBC), biliary atresia (BA) [60,61,62,63], alcoholic liver disease (ALD), and metabolic dysfunction-associated liver disease (MASLD) [10,60,62]. Both processes are activated by liver damage and, with their chronic progression, instead of promoting tissue repair, they contribute to the progression of the resulting diseases within the organ [60,61,62,63,64,65]. They jointly involve a group of cells, including, among others, hepatic stellate cells (HSCs), hepatocytes, hepatic progenitor cells (HPCs), mesenchymal stem cells, and immune cells [10,60,61,63,64,65].

Clear correlations between these processes have been demonstrated [10,60,61,63,64,65,66], although the order in which they occur remains unclear [61]. In scientific studies, bile duct markers such as cytokeratin 7 (CK7) and cytokeratin 19 (CK19) were used [10,65,67,68]. Glutamine synthetase (GS) was used as a marker of regenerative processes to compare the activity of cytokeratins. In chronic diseases, elongation and increased structural complexity of the DR were observed, accompanied by heightened expression of CK7/CK19 and minimal expression of GS [63]. The intensification of both the DR and fibrosis negatively affected liver pathology and always correlated with the degree of liver damage [10,60,61,63,66].

### 4.1. Ductular Reaction-Driven Extracellular Matrix Deposition

The DR is a process that generates both positive and negative effects [10,60,62,64,65,66]. It is characterised by hyperproliferation of ductular structures consisting mainly of cholangiocytes and HPCs, which are mobilised [60,61,62,63,64,65], and, depending on the type of injury, HPCs may differentiate into hepatocytes to repair damaged tissue or play a role in HSC activation and the infiltration of inflammatory cells in DR, which is clearly associated with the progression of liver damage and the development of cirrhosis and tumours. [10,60,61]. When exposed to chronic damage, cholangiocytes and HPCs undergo senescence, adopting a secretory phenotype [60,63]. They begin to secrete cholangiokinins—substances similar to those produced by inflammatory cells [60]. These include cytokines, chemokines, and growth factors [9,10,60,61,62,63]. The main agents involved here are interleukin-6 and transforming growth factor-β1. Transforming growth factor-β1 promotes cholangiocyte proliferation and the activation of hepatic stellate cells, leading to ECM accumulation and liver fibrosis. Interleukin-6 also promotes cholangiocyte proliferation and activates macrophages, leading to a ductular reaction and hepatitis [62,63]. HPCs are also a source of PDGF, CTGF, and Hedgehog ligands, which are believed to act as fibrogenic factors [10]. The function of these substances is to facilitate communication among liver cells, recruit immune cells, and coordinate interactions between reactive ductal cells and portal myofibroblasts [60,61,62,63]. In this way, HPCs, along with hepatocytes and cholangiocytes, can undergo epithelia-mesenchymal transition (EMT), a process of cellular reprogramming from an epithelial to a mesenchymal phenotype, thereby exacerbating fibrosis [10,65].

Activated HSCs transdifferentiate into myofibroblasts, secreting ECM components, collagen deposits, and cytokines that drive the proliferation and differentiation of liver cells. Among the cytokines secreted by Ito cells are HGF, lymphotoxin-beta, FGF, IL-6, NOTCH, non-canonical Notch ligand delta-like 1, and TGF-β1. HSCs regulate HPC proliferation through the antiproliferative action of TGF-β1. In this way, HSCs not only promote fibrosis but also modulate HPC activity, influencing the balance between regeneration and processes leading to progressive liver damage. Excessive synthesis of ECM components by activated HSCs inhibits hepatocyte proliferation. The lack of a beneficial effect of MMP-phenotype macrophages on connective tissue repair results in disruption of the liver’s vascular architecture and, consequently, the development of cirrhosis [66]. DR cells also secrete angiogenic factors, including VEGF-A, PDGF-B, TGF-β2, and endothelin-1, interacting with myofibroblasts in the remodelling of the bile ducts and further exacerbating liver fibrosis [60].

### 4.2. Extracellular Matrix-Driven Modulation of Ductular Reaction

Based on histopathological features, Desmet classified DR into four distinct types. Type 1 (typical) DR remains confined to the portal area and originates from pre-existing cholangiocytes with minimal reorganisation of the ductal structure. This type is reversible and does not establish any new canaliculo-ductal connections. Type 2A DR occurs in periportal areas, as seen in chronic cholestatic liver diseases, involving HPCs and cells derived from reprogrammed hepatocytes. Type 2B DR occurs in central perinuclear areas and is induced by hypoxia. And the final type 3 (pathological) is caused by the activation and proliferation of HPCs located in Hering’s ducts in response to massive hepatocyte necrosis [61,62]. Type 2A, type 2B, and type 3 DRs form canalicular-forming connections and thus potentially participate in bile drainage and clear hepatocyte damage in chronic diseases. Based on these characteristics, DRs are classified into non-invasive and invasive types. Non-invasive DR is also known as type 1 or typical DR, while invasive DR is referred to as type 2 or 3 or atypical DR. Invasive DRs are characterised by intense proliferation of ductal structures that infiltrate the hepatic lobules, accompanied by activation and an increased number of HPCs. These phenomena coexist with extensive hepatocyte necrosis, significant loss of parenchyma, and progressive fibrosis, as well as changes with the potential for cellular transformation. This type of DR has been described in patients with chronic cholestatic diseases [62].

The hepatic portal zone is supported by a network of extracellular matrix (ECM) proteins that form what is known as Mall’s space. Rich in collagen, fibronectin, proteoglycans, and laminin, this network provides tensile strength to the portal space and helps maintain its structural integrity [10,61]. Thanks to this rich microenvironment and the stiffness it provides, HPCs located in the periportal zone can be activated in response to present damaging factors, both physical and biochemical. The ECM thus serves as a scaffold conducive to HPC activation and migration during liver regeneration, with laminin and collagen IV supporting their self-renewal [61], while fibronectin and collagen promote cell cycle arrest and the differentiation of HPCs into hepatocytes [61,64]. Chronic liver diseases lead to changes in structural stiffness. There is an accumulation of collagen fibres, mainly type 1, in the ECM. The YAP protein responds to these stiffness changes, enabling liver progenitor cells to interpret mechanical stimuli and adjust their proliferative response [61]. Initially, increasing stiffness has a positive effect on HPCs, leading to their activation and proliferation; however, once a certain elasticity threshold is exceeded, an excessively stiff ECM inhibits their expansion, limits regenerative capacity, and promotes a pro-inflammatory phenotype [60]. The DR response becomes ineffective and further promotes ongoing chronic liver damage through activated HPC [10].

Together, these reciprocal interactions indicate that the DR–ECM interface is not merely a coexistence of two histological abnormalities but a self-amplifying remodelling niche in which attempted epithelial repair is progressively converted into fibrogenesis, architectural distortion, and impaired parenchymal regeneration.

## 5. Parenchymal Extinction as an Indicator of Architectural Loss

### 5.1. Vascular Injury and Hepatocyte Loss

Parenchymal extinction signifies the focal loss of hepatocytes and adjacent microvascular structures. The basis of this process is vascular injury, including sinusoidal endothelial cell damage and vascular obstruction, associated with fluid translocation into vessel walls and interstitial tissue, causing vascular compression [60]. Damage to sinusoidal endothelial cells promotes the formation of blood clots, as the expression of thrombomodulin, nitric oxide and prostaglandin I2 is weakened, while the exposure of von Willebrand factor, integrins and other receptors that interact with activated platelets is increased [62,65]. Vascular obstruction begins in small hepatic veins and over time, involves larger branches [60]. The formation of microthrombi is also a response to inflammation. In this situation, immune cells and fibroblasts participate in coagulation, which means that the presence of microthrombi is also a factor that further exacerbates inflammation [65]. Vascular injury leads to hypoxia of the hepatocytes [10]. Even early changes in blood supply cause stress zones, visible as variations in the colour of the hepatocyte cytoplasm within the lobules, as well as atrophy [60]. Hepatocytes are also exposed to stress caused by the chronic portal and lobular inflammation. As a result, the basic structural units of the liver parenchyma become damaged and undergo apoptosis [10]. When the vascular obstruction involves larger vascular branches, the areas of hepatocyte loss expand, leading to architectural collapse. The portal tracts are no longer surrounded by hepatocytes, but by a collapsed reticulin network [60]. Activated by inflammation, macrophages and myofibroblasts lead to excessive collagen accumulation in the extracellular matrix, causing septal formation and separating hepatic parenchyma into nodules, resulting in an image visible in cirrhosis [62,66]. The nodules contain inflammatory cells, ductal proliferation and edema, which causes the central hepatic veins to be compressed and thrombotic and may lead to their atrophy, accompanied by the formation of vascular shunts [63]. Microvascular thrombosis also gets replaced by fibrous tissue, increasing congestion [65].

Lesions may occur simultaneously at different stages. The vascular injury underlies this process, yet hepatocyte loss is easier to assess and analyse during histological examination in the context of cirrhosis progression and regression [60].

### 5.2. Architectural Collapse and Incomplete Regression

Parenchymal regeneration, as well as regression of fibrosis and clinical improvement, is possible if the damaging factor is eliminated or suppressed. In the process of fibrosis regression, the septa become progressively thinner until they are perforated by proliferating hepatocytes derived from progenitor cells. What remains of the septa are delicate periportal spikes in the portal tracts [10]. Remodelling of the parenchymal extinction areas may result in similar to normal appearance, yet it is connected with not only proliferating hepatocytes but also myofibroblasts, inflammatory cells, and proliferating ductules [63]. The proliferating bile ductules form complex networks with numerous loops. The growth of newly formed hepatocyte nodules is disorganised, as there is no basement membrane scaffold. The architecture is so distorted that regeneration does not lead to the formation of correct structures. Additionally, growing regenerative nodules may compress the hepatic veins, which only further exacerbates parenchymal ischaemia and triggers apoptosis, fibrosis, and another attempt at regeneration [64]. Regression does not equal reversal. Other than that, the large parenchymal extinction lesions are not prone to regeneration and may persist [63]. Furthermore, even if the changes visible in cirrhosis regress, mutations caused by the damaging factor remain in the hepatocytes. In the cycles of parenchymal extinction and regeneration, the expression of vascular endothelial growth factor is increased, which leads to genomic instability. This means that the risk of hepatocellular carcinoma remains higher than normal. In addition, if the damaging factor affects the liver for a sufficiently long time, vascular remodelling resulting from compression and thrombosis may also be irreversible. What remains within the portal tracts are the bile duct and hepatic artery, with no visible portal vein [10]. The practical histopathological features used to assess ductular reaction, extracellular matrix remodelling, and parenchymal extinction are summarised in Table 6.

### 5.3. Tumour-like Presentation and Hepatocarcinogenic Context

Parenchymal extinction and hepatocellular carcinoma are connected because the former process increases the risk of carcinogenesis [10]. On the other hand, there is a case study showing that, although parenchymal extinction and hepatocellular carcinoma can be differentiated through histological examination, the clinical picture and imaging results alone can be similar. The case concerns a 47-year-old patient with chronic hepatitis B infection and Child-Pugh class A cirrhosis who had elevated alpha-fetoprotein and other liver parameters such as aspartate aminotransferase and alanine aminotransferase. Abdominal ultrasonography revealed a 3-cm-sized hypoechoic lesion that, in a T1-weighted MRI sequence, was hyperintense visible in the arterial phase, isointense in the delayed phase, and hypointense in the hepatobiliary phase. The clinical picture suggested hepatocellular carcinoma, but histological examination of the removed lesion revealed features characteristic of parenchymal extinction without the presence of cancer cells and with a negative result for the hepatocellular carcinoma diagnostic marker glypican-3 [61].

### 5.4. Integration with Ductular Reaction and Extracellular Matrix Remodelling

**Figure 1:**
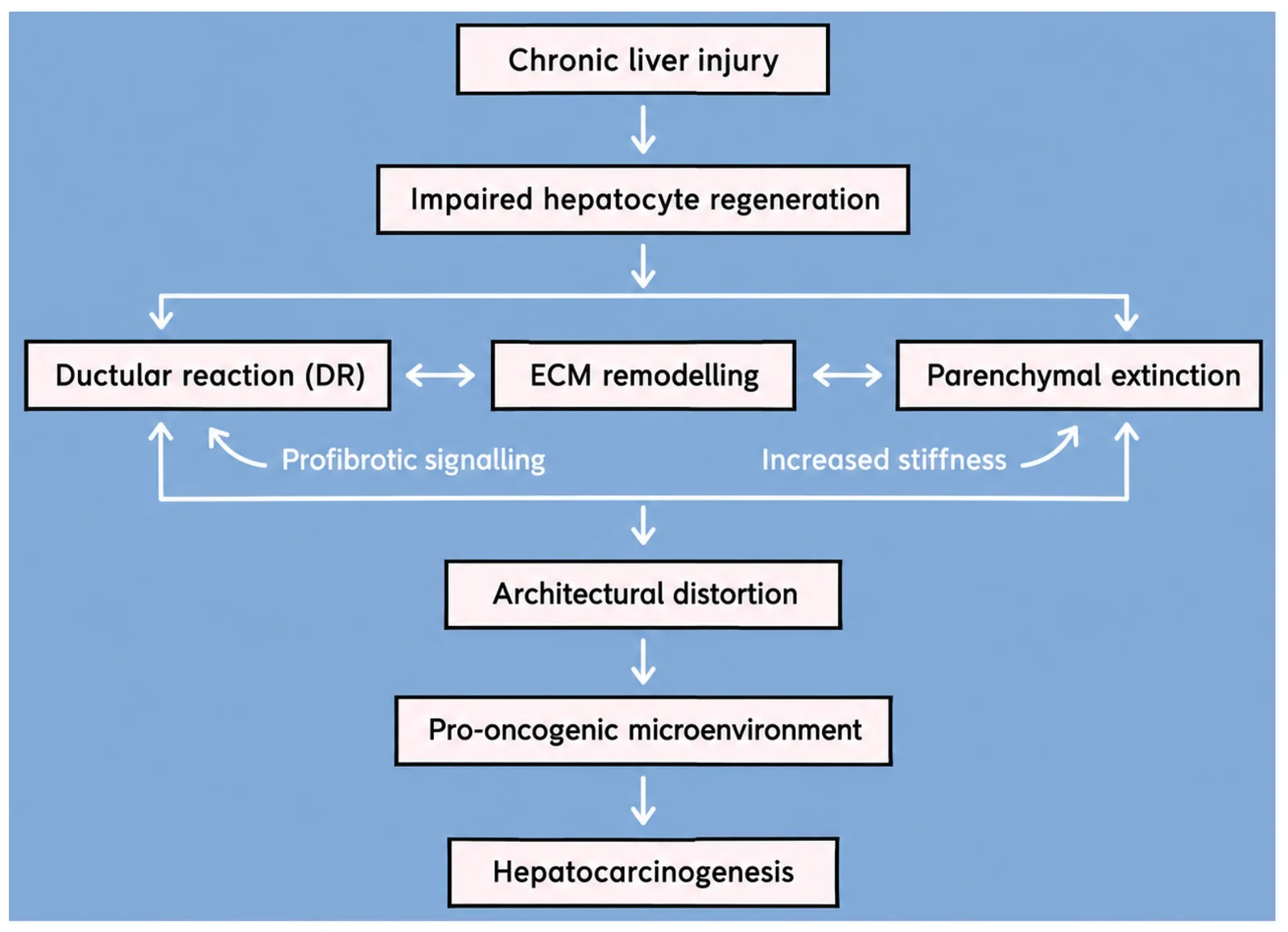
Schematic summary of the interactions between DR, ECM remodelling, and parenchymal extinction.

Parenchymal extinction is linked to ductular reaction and extracellular matrix remodelling, as all of them are part of the response to damaging factors. These three processes coexist, but also reinforce one another, exacerbating pathological repair rather than healthy regeneration, which drives disease progression towards irreversible changes. Chronic inflammation and damaging factors leading to hepatocyte apoptosis and formation of parenchymal extinction lesions are also activating factors for hepatic stellate cells, which are responsible for the excessive deposition of extracellular matrix proteins. Excessive ECM accumulation also disrupts liver architecture, maintaining the collapse caused by hepatocyte loss [70,71]. The situation is similar to ductular reaction. The proliferation and differentiation of hepatic progenitor cells leads to the formation of reactive ductules within the portal area and in the liver parenchyma as a pathological attempt to regenerate the liver structure lost during parenchymal extinction [28]. A self-sustaining cycle develops, leading to progressive inflammation, fibrosis and carcinogenesis (Figure 1).

## 6. Hepatocarcinogenesis as the Outcome of Maladaptive Remodelling

Persistent hepatic injury promotes chronic maladaptive remodelling, including ductular reaction, extracellular matrix remodelling, and the parenchymal extinction sequence. Together, these processes establish a pro-inflammatory, profibrogenic, and immunosuppressive microenvironment characteristic of fibrotic and cirrhotic livers, in which most cases of HCC arise [72].

### 6.1. DR and HCC

Ductular reaction is a complex cellular response characterised by the proliferation of reactive bile ducts induced by various liver injuries [26,28]. Reactive ductule cells (RDCs) demonstrate a bipotential biliary/hepatic progenitor cell (HPC) phenotype [71,73]. A study by Qianhui Xu et al. identified three functionally distinct cholangiocyte subpopulations within the peritumoural DR ecosystem: stem-like, hepatocyte-like, and small-duct type cholangiocytes [73]. Small-duct-type cholangiocytes interact with matrix cancer-associated fibroblasts (mCAFs), suggesting association between matrix remodelling and ductular reaction [73]. DR cells actively modulate the hepatic microenvironment by secreting various cytokines and chemokines [28,73]. Moreover, it was found that the tumour margin region is enriched with cholic acid, which promotes an immunosuppressive environment by activating the NR1H4 receptor, thereby upregulating PD1 expression and leading to CD8+ T cell dysfunction, facilitating tumour escape [73]. DAMPs, particularly HMGB1 released from necrotic hepatocytes, have been reported to activate the DR and promote malignant transformation of adjacent cells; however, further research is needed to elucidate the exact underlying mechanisms [74]. The igh proliferative index of DR is correlated with both poor overall survival and shorter disease-free survival after resection [72,73]. 75 Quantitative AI-based analysis reveals that DR scores progressively decrease with distance from the tumour margin, and high scores correlate significantly with advanced tumour stage, higher recurrence rates and resistance to anti-PD1 immunotherapy [73].

### 6.2. ECM Remodelling and HCC

It has been found that ECM stiffness and the tumour microenvironment (TME) interactions play a crucial role in the initiation, progression and immunotherapy of HCC [75,76]. ECM remodelling increases tissue stiffness through excessive deposition and cross-linking of collagen, fibronectin, laminins, and proteoglycans. Depending on the method of assessment, fibrotic and cirrhotic liver tissue demonstrates substantially higher stiffness than healthy liver tissue [75,77]. Syndecan-1 and agrin (PGs) are overexpressed in patients with cirrhosis and HCC [75,78,79]. Moreover, tumour-secreted Argin enhances the stability of vascular endothelial growth factor receptor 2, making HCC highly vascularised [80]. Lysyl oxidase (LOX), involved in forming cross-linked collagen, is also elevated in patients with fibrosis and HCC and correlates with poor prognosis [81]. Matrix stiffness-related protein DYNLL1 promotes HCC cell progression and migration via the Wnt/beta-catenin pathway. ECM stiffness impedes immune cell infiltration into the TME and worsens the efficacy of tumour-targeted ICIs. HSCs activated during ECM remodelling can also differentiate into cancer-associated fibroblasts (CAFs). Collagen type I alpha1, collagen type III alpha 1 and LOX are markers related to CAF development. The YAP-1-positive CAF subpopulation promotes HCC progression by activating signalling pathways such as autophagy through up-regulating matrix stiffness-related genes. TGF-beta secreted by HCC cells induces CLFC1 production by CAFs which increases TGF-beta secretion, creating a loopback and accelerating HCC progression. CAFs also play a role in enabling cancer cell invasion by generating physical trajectories in the ECM [75].

### 6.3. Parenchymal Extinction and HCC

Parenchymal extinction is defined by Wanless as a region with focal loss of contiguous hepatocytes and adjacent microvascular structures [60]. The pathogenesis starts with vascular injury which leads to vascular obstruction, causing hepatocellular ischaemia, resulting in PE lesions with collapse of architecture [60]. Chronic ischaemia creates a hypoxic carcinogenic microenvironment which stimulates neovascularisation and activates specific signalling pathways such as HIF1α and LOXL2 pathways, AMPK/mTOR and autophagy signalling, enabling the survival of transformed cells [74]. Enduring oxidative stress, often stemming from mitochondrial dysfunction in ischaemic areas, contributes to cellular senescence and genomic instability, further facilitating the transition from cirrhosis to HCC [82]. Vascular injury disrupts blood flow balance– hepatic artery inflow exceeds the system’s outflow capacity, leading to endothelial leak, transudation and edema within vessel walls [10]. It further compresses adjacent veins, exacerbating the ischaemic state [60]. The capillarisation of sinusoids and formation of vascular shunts contribute to abnormal blood flow characteristic for the premalignant environment [60,74]. Impairment of the local microvasculature also delays repair mechanisms, so that tissue replacement from progenitor cells becomes the dominant pathway to repopulate regions of injury [60]. Regenerative nodules are clonal and lack common mutations. They grow in a disorganised manner, further compressing hepatic veins and contributing to the cycle of congestion, necrosis and scarring [65]. This structural transformation creates a pro-oncogenic niche, as the majority of HCCs occur in fibrotic/cirrhotic livers [83]. Continuous regenerative pressure, combined with chronic inflammation and the secretion of mitogenic factors like HGF and epiregulin by activated hepatic stellate cells, drives the malignant transformation of hepatocytes toward tumour-initiating cells [82,83].

### 6.4. Summary

Ductular reaction, extracellular matrix remodelling, and parenchymal extinction interact with one another and collectively transform chronically injured liver tissue into a possibly pro-oncogenic microenvironment. Parenchymal extinction, initiated by vascular obstruction and ischaemic hepatocyte loss, distorts the hepatic architecture and promotes regenerative pressure. This architectural collapse is accompanied by ductular reaction, which contributes to inflammatory and immunosuppressive niche formation. In parallel, extracellular matrix remodelling increases tissue stiffness and activates mechanotransduction pathways, including YAP/TAZ and Wnt/β-catenin signalling, thereby supporting cancer cell invasion and tumour progression. Ultimately, these three maladaptive remodelling mechanisms promote chronic inflammation, genomic instability, immune dysfunction, and microenvironmental conditions that favour hepatocellular carcinoma initiation and progression.

To integrate the mechanisms discussed above, ductular reaction, extracellular matrix remodelling, and parenchymal extinction can be interpreted as three complementary histopathology-anchored endpoints of maladaptive liver regeneration. Their key morphological, cellular, functional, and carcinogenic implications are summarised in Table 7.

Therefore, these endpoints should not be assessed as isolated lesions, but as interconnected components of a progressive remodelling continuum linking failed regeneration, fibrosis, vascular injury, and hepatocarcinogenesis.

## 7. Conclusions

Maladaptive liver regeneration in chronic liver disease should be interpreted not as a single isolated process, but as an integrated histopathological continuum involving ductular reaction, extracellular matrix remodelling, and parenchymal extinction. These endpoints reflect the progressive failure of adaptive repair, in which regenerative mechanisms become chronically activated, spatially disorganised, and functionally insufficient. Their interaction creates a self-perpetuating cycle of inflammation, fibrogenesis, vascular injury, architectural collapse, and impaired hepatocyte regeneration.

The combined evaluation of ductular reaction, ECM remodelling and parenchymal extinction may provide a more comprehensive framework for assessment of disease progression and carcinogenic risk than fibrosis staging alone, from a histopathological perspective. At present, this framework should therefore be regarded as a pathology-orientated, hypothesis-generating model rather than a validated composite scoring system. Further studies are needed to define reproducible scoring systems, validate molecular and immunohistochemical markers, and clarify the incorporation of these endpoints with non-invasive biomarkers and clinical risk stratification in chronic liver disease.

## Figures and Tables

**Table 1:** Types of ductular reaction according to Desmet’s classification [25].

Type of DR	Mechanism of Formation	Morphological Features	Typical Clinical Situations/Etiology
Type 1—typical reaction	Proliferation of pre-existing cholangiocytes	Bile ductules undergo elongation and branching, and their lumen dilates, which allows their adaptation to edema and inflammation of the portal tract mesenchyme	Acute complete biliary duct (BD) obstruction, alpha-naphthyl isothiocyanate intoxication, cytokine stimulation (e.g., interleukin 6)
Type 2A—atypical reaction	Ductular metaplasia of mature hepatocytes, most commonly in periportal areas	Formation of ductule-like structures from hepatocytes	Chronic cholestasis—accumulated bile acids are promoting the process of periductular fibrosis and metaplasia
Type 2B—atypical reaction	Hypoxia-induced ductular metaplasia	Microscopic picture like type 2A; accompanying fibrosis	Centrilobular areas of parenchymal hypoxia
Type 3—invasive reaction	Activation and proliferation of liver progenitor cells (LPCs) residing in the canals of Hering	HPCs differentiate into both hepatocytes and biliary tract cells. Anastomosing labyrinthine proliferation of ductules extending beyond the limiting plate into the lobule	Massive loss of parenchymal cells and severe parenchymal injury

**Table 2:** Immunohistochemical classification of DR [35].

DR Type	Immunohistochemical Profile
Primitive type	CD56+/EMA−/CD10−
Differentiating type	CD56+/EMA+/CD10+
Obstructive type	CD56−/EMA+/CD10−

**Table 3:** Multifactorial etiopathogenesis of DR and mechanisms of its induction.

Factor	Mechanism/Description	Context
Portal perfusion disturbances and tissue hypoxia [40]	Process mediated by the activation of the YAP/CTGF axis, promotes the progression of fibrotic changes	Occurs during NASH; the clinical manifestation of the hepatic arterial buffer response (HABR) in patients with NAFLD is associated with deeper organ damage and higher susceptibility to metabolic disorders
Western diet [41]	In the *CGI-58* LivKO mouse model, it provoked exacerbated DR	May influence the course of NASH and fibrosis
Benzo[a]pyrene in tobacco smoke [42]	Promotes DR through the induction of ferroptosis in biliary epithelial cells	Toxic factor
Dense infiltration of IgG4+ plasma cells [43]	Accompanied by exacerbated DR which correlates with disease progression	Occurs in autoimmune hepatitis (AIH); DR as a presumed cause of advanced organ damage requires further verification in prospective studies

**Table 4:** Characteristics of the signalling network and cellular interactions determine the course of the ductular reaction (DR) and organ fibrosis.

Mechanism Category	Pathway/Factor	Role and Impact on DR Pathogenesis
Signalling pathways of HPCs and cholangiocytes	Notch pathway [28]	Determines the differentiation of HPCs towards cholangiocytes; in models of steatohepatitis, its inhibition reduces DR and cholestatic liver fibrosis
	Wnt/β-catenin and Hedgehog (Hh) [28]	Stimulate the proliferation, expansion, and survival of HPCs; the Hh pathway is particularly active in the fibroproliferative response in PBC
	Hippo pathway (YAP/TAZ) [28]	Initiates transcriptional programs necessary to trigger DR; enables the differentiation of HPCs into cholangiocytes
	HGF/c-MET axis and mTOR [28]	Support the migratory phenotype of cells, determining phenotypic plasticity and adaptive tissue remodelling.
	NF-κB-inducing kinase (NIK) [44,45]	Promotes pathological DR and cholangiokine secretion; exacerbates fibrosis and impairs regeneration
Microenvironment and immune cells	Ductular reaction-associated neutrophils (DRANs) [46]	They form neutrophil extracellular traps (NETs) and release elastase; stimulate DR expansion, fibrosis, and angiogenesis
	MAIT cells (Amphiregulin—AREG) [47]	They stimulate proliferation and abnormal differentiation of cholangiocytes; induce a profibrogenic environment through epithelial mesenchymal crosstalk
Survival and metabolic mechanisms	Autophagy (Atg5/Atg7) and mTORC1 [48]	Accumulation of p62 protein (in autophagy deficiency) activates the Nrf2 pathway; p62 limits uncontrolled DR and prevents neoplastic transformation
	TRAIL signalling [49]	Deficiency in proapoptotic signalling through the TRAIL receptor decreases cell loss; leads to the pathological accumulation of DR and bridging fibrosis

**Table 5:** Molecular targets and potential therapeutic strategies in the modulation of DR and liver fibrosis.

Molecular Target/Signalling Pathway	Role in DR Pathogenesis and Fibrosis	Promising Therapeutic Strategy/Intervention
Mevalonate pathway (MVA) [50]	Maintains the reactive cholangiocyte phenotype through intracellular accumulation of cholesterol and geranylgeranyl pyrophosphate.	Inhibition of the pathway with statins can lead to the suppression of pathological DR expansion.
Vitamin D/TXNIP axis [51]	TXNIP deficiency increases TNF-α and TGF-β secretion by cholangiocytes, activating Kupffer cells and hepatic stellate cells (HSCs), leading to increased inflammation and collagen deposition.	Induction of TXNIP expression in ductular cells via the active form of vitamin D (hepatoprotective effect).
Sortilin (LIF/YAP/Vangl2 axis) [52]	Promotes fibrosis through the secretion of mediators that activate HSCs and stimulate collagen deposition.	Selective blocking of the sortilin receptor in the ductular population.
RAGE receptor [53]	Acts as a master regulator promoting a profibrotic environment via Jagged1 secretion and activation of HSCs and portal fibroblasts in trans.	Blocking the RAGE axis to prevent extracellular matrix deposition.
Gli1 protein/Hedgehog pathway [54,55]	Gli1 protein mediates the activation and differentiation of HPCs into reactive cholangiocytes (with simultaneous activation of Shh and Yap axes).	Application of pathway inhibitors (e.g., GANT61), mitigating injury and inhibiting DR.
Numb protein/Notch pathway [56]	Numb is a suppressor of the Notch pathway—it inhibits undesirable HPC differentiation toward cholangiocytes and promotes their conversion into functional hepatocytes.	Gene therapy that involves supplementation with exon 3 of the *Numb* gene (especially in PBC and cholestasis).
Extracellular matrix protein 1 (ECM1) [57]	Binds profibrotic factors and limits integrin αvβ6-dependent TGF-β activation; its levels decrease in chronic liver injury.	Restoration of physiological ECM1 expression, which inhibits the induction of DR.
Granulocyte colony-stimulating factor (G-CSF) [58]	Under cholestatic conditions, pathological DR promotes periportal fibrosis.	G-CSF administration to mobilize bone marrow stem cells and induce HGF expression, which inhibits DR and fibroblasts, leading to the alleviation of periportal fibrosis and improved survival.
Natural compounds: *Astragalus* saponins (Astragaloside I, Cycloastragenol) [59]	Reduces the expression of CK19 and α-SMA markers.	Inhibition of DR and fibrosis.
Bile acid receptors and paracrine pathways (NAFLD) [11]	Promoting inflammation and DR through specific signalling axes in the biliary microenvironment.	Blocking S1PR2 receptors and inhibiting histamine and secretin signalling.

**Table 6:** Practical histopathological assessment of ductular reaction, extracellular matrix remodelling and parenchymal extinction.

Endpoint	Minimum Morphological Features on H&E	Typical Distribution	Recommended Markers	Key Differential Diagnoses and Pitfalls	Practical Grading	Suitable Specimen Types
Ductular reaction (DR)	Increased number of small ductular structures, usually lined by cuboidal epithelial cells; ductular proliferation may show branching, elongation and irregular profiles. Neutrophils or other inflammatory cells and periportal fibrosis may accompany the reaction [9,67].	Predominantly portal and periportal; in more extensive reactions, ductular structures may extend into the adjacent parenchyma [9,67].	H&E for morphology; CK7 and CK19 to highlight biliary structures, particularly when reaction is inconspicuous on H&E [9,68].	Biliary obstruction, PBC, PSC, DILI and other biliary-pattern injuries may produce overlapping ductular changes. Interpretation should incorporate clinical context and associated findings. CK7/CK19 identify a biliary phenotype but are not progenitor-specific and do not establish lineage [9,67,68].	No universally accepted grading system across all liver diseases. Assessment should consider extent, density, complexity and parenchymal extension of DR; qualitative or semi-quantitative assessment may be used [9].	Liver needle biopsy containing adequate portal tracts; resection specimens allow more comprehensive assessment of spatial distribution [9,67].
ECM remodelling	Portal or periportal fibrous expansion, perisinusoidal fibrosis, fibrous septa and bridging fibrosis; advanced fibrosis is associated with architectural distortion and cirrhosis [13,14].	Distribution depends on aetiology and stage; may be portal perisinusoidal or septal ging [13,14].	H&E for architecture; Masson’s trichrome and Sirius Red for collagen; reticulin for assessment of the hepatic framework and hepatocyte plates. Digital image analysis of Sirius Red can be used for collagen proportionate area (CPA) [13,14].	Fibrosis should be distinguished from collapse, congestion and other architectural alterations. Histological fibrosis stage should not be considered identical to direct quantitative collagen measurement. CPA provides a continuous measure of collagen content but does not fully capture architectural information [13,14,69].	Disease-specific semi-quantitative staging systems should be used according to aetiology. When collagen quantity is the endpoint, CPA/digital image analysis provides a quantitative measure [69].	Adequate liver biopsy is suitable for fibrosis staging; larger resection specimens provide broader assessment of the spatial distribution and heterogeneity of ECM remodelling [13,69].
Parenchymal extinction (PE)	Focal loss of contiguous hepatocytes with collapse or distortion of hepatic architecture; lesions may show replacement by fibrous tissue, altered sinusoidal architecture and vascular abnormalities. Approximation of portal and hepatic venous structures may occur [61,64].	Often associated with areas of vascular injury, ischemia and microvascular compromise; in advanced cirrhosis, lesions may contribute to architectural collapse and septal formation [61,64].	H&E for hepatocyte loss and architectural collapse; trichrome can demonstrate associated fibrous replacement; reticulin may assist in assessing collapse of the hepatic framework. Additional vascular assessment may be required when vascular obstruction is suspected [61,64].	Confluent necrosis, ischemic injury, fibrotic scars, regenerative collapse and some mass-like lesions may overlap morphologically. In cirrhotic nodules, PE may mimic HCC radiologically; correlation with morphology and appropriate IHC may be required [61].	No universally validated routine grading system. Assessment should be based on the presence, number, extent and distribution of lesions, together with the associated vascular and architectural changes [61,64].	Resection and explant specimens are particularly informative because they preserve larger areas of architecture. Needle biopsies can demonstrate PE but may underestimate focal lesions because of sampling limitations [61].

**Table 7:** Practical histopathological assessment of maladaptive liver regeneration endpoints.

Endpoint	Main Histological Features	Useful Stains/Markers	Clinicopathological Relevance and Link to HCC
Ductular reaction	Irregular ductular profiles at the portal–parenchymal interface, with periportal expansion and possible lobular extension in advanced injury [9,26,28,35].	CK7, CK19; EpCAM and SOX9 for a progenitor-associated phenotype; CD56, EMA, and CD10 in selected cases [25,27,36]	Reflects impaired hepatocyte regeneration and activation of an epithelial–fibrogenic niche. In the peritumoral setting, proliferative DR has been associated with HCC recurrence and poorer response to anti-PD-1 therapy [71,73].
ECM remodelling	Portal, periportal, or perisinusoidal matrix deposition progressing to fibrous septa, bridging fibrosis, and architectural distortion [10,63,70].	Trichrome or Sirius red for collagen; reticulin for framework alterations; α-SMA for activated myofibroblastic cells [10,24,62,63].	Contributes to architectural distortion and impaired regeneration. Increased matrix stiffness, collagen cross-linking, CAF activation, and immune exclusion may support HCC progression [75,76,81].
Parenchymal extinction	Contiguous hepatocyte loss with adjacent microvascular loss, reticulin collapse, vascular obliteration, and fibrous replacement [10,60,63].	Reticulin and trichrome stains; glutamine synthetase and vascular or ductular markers in selected cases [61,62].	Represents vascular–parenchymal loss with incomplete reversibility. Associated hypoxia, microvascular injury, oxidative stress, and repeated regenerative pressure may contribute to persistent hepatocarcinogenic risk [10,60,65,82].

## Data Availability

No new data were created or analyzed in this study. Data sharing is not applicable to this article.

## Abbreviations

The following abbreviations are used in this manuscript:

ACLD	advanced chronic liver disease
ADMSCs	adipose-derived mesenchymal stem cells
AI	artificial intelligence
AIH	autoimmune hepatitis
ALD	alcohol-associated liver disease
AMPK	AMP-activated protein kinase
ANG-2	angiopoietin-2
APRI	aspartate aminotransferase-to-platelet ratio index
AREG	amphiregulin
AST	aspartate aminotransferase
Atg5/Atg7	autophagy-related proteins 5 and 7
BA	biliary atresia
BD	bile duct
BM	basement membrane
BMSCs	bone marrow-derived mesenchymal stem cells
CAFs	cancer-associated fibroblasts
CBDO	common bile duct occlusion
CD	cluster of differentiation
c-MET	mesenchymal–epithelial transition factor receptor
CGI-58	comparative gene identification-58
CHB	chronic hepatitis B
CK7/CK19	cytokeratins 7 and 19
CLD	chronic liver disease
CLCF1	cardiotrophin-like cytokine factor 1
COL1A1	collagen type I alpha 1 chain
CTGF	connective tissue growth factor
CXCR2	C-X-C motif chemokine receptor 2
CYFRA21-1	cytokeratin 19 fragment antigen 21-1
DAMPs	damage-associated molecular patterns
DNA	deoxyribonucleic acid
DR	ductular reaction
DRANs	ductular reaction-associated neutrophils
DYNLL1	dynein light chain LC8-type 1
ECM	extracellular matrix
ECM1	extracellular matrix protein 1
ELF	Enhanced Liver Fibrosis score
EMA	epithelial membrane antigen
EMT	epithelial–mesenchymal transition
EpCAM	epithelial cell adhesion molecule
ESCs	embryonic stem cells
FGF	fibroblast growth factor
FIB-4	Fibrosis-4 index
G-CSF	granulocyte colony-stimulating factor
Gal	galanin
Gli1	GLI family zinc finger 1
GS	glutamine synthetase
HABR	hepatic arterial buffer response
HBV	hepatitis B virus
HCC	hepatocellular carcinoma
HCV	hepatitis C virus
HGF	hepatocyte growth factor
Hh	Hedgehog
HIF-1α	hypoxia-inducible factor 1 alpha
HMGB1	high-mobility group box 1
HPCs	hepatic progenitor cells
HSCs	hepatic stellate cells
ICAM-1	intercellular adhesion molecule 1
ICIs	immune checkpoint inhibitors
IgG4	immunoglobulin G4
IL-6	interleukin-6
IL-8	interleukin-8
IM	interstitial matrix
KCs	Kupffer cells
KRT7	keratin 7
LCN2	lipocalin 2
LIF	leukaemia inhibitory factor
LIHC	liver hepatocellular carcinoma
LivKO	liver-specific knockout
LOX	lysyl oxidase
LOXL2	lysyl oxidase-like 2
LSECs	liver sinusoidal endothelial cells
MAIT	mucosal-associated invariant T cells
MASH	metabolic dysfunction-associated steatohepatitis
MASLD	metabolic dysfunction-associated steatotic liver disease
MDR2	multidrug resistance protein 2
MELD	Model for End-Stage Liver Disease
mCAFs	matrix cancer-associated fibroblasts
MMPs	matrix metalloproteinases
MoMFs	monocyte-derived macrophages
MRI	magnetic resonance imaging
MSCs	mesenchymal stem cells
mtDNA	mitochondrial DNA
mTOR	mechanistic target of rapamycin
mTORC1	mechanistic target of rapamycin complex 1
MVA	mevalonate pathway
NAFLD	non-alcoholic fatty liver disease
NASH	non-alcoholic steatohepatitis
NCAM	neural cell adhesion molecule
NETs	neutrophil extracellular traps
NF-κB	nuclear factor kappa B
NIK	NF-κB-inducing kinase
NLRP3	NLR family pyrin domain-containing 3
NR1H4	nuclear receptor subfamily 1 group H member 4
Nrf2	nuclear factor erythroid 2-related factor 2
p62/SQSTM1	sequestosome 1
PAMPs	pathogen-associated molecular patterns
PBC	primary biliary cholangitis
PD-1	programmed cell death protein 1
PDGF	platelet-derived growth factor
PDGF-B	platelet-derived growth factor subunit B
PE	parenchymal extinction
PGs	proteoglycans
PRO-C3	N-terminal type III collagen propeptide
PSC	primary sclerosing cholangitis
RAGE	receptor for advanced glycation end products
RDCs	reactive ductular cells
REDD1	regulated in development and DNA damage responses 1
RNA	ribonucleic acid
ROBO1	roundabout guidance receptor 1
ROS	reactive oxygen species
S1PR2	sphingosine-1-phosphate receptor 2
Shh	Sonic hedgehog
SLIT2	slit guidance ligand 2
SOX9	SRY-box transcription factor 9
α-SMA	alpha-smooth muscle actin
TAZ	transcriptional coactivator with PDZ-binding motif
TGF-β	transforming growth factor beta
TIMPs	tissue inhibitors of metalloproteinases
TLRs	Toll-like receptors
TME	tumour microenvironment
TNF-α	tumour necrosis factor alpha
TRAIL	TNF-related apoptosis-inducing ligand
TXNIP	thioredoxin-interacting protein
Vangl2	Van Gogh-like 2
VCAM-1	vascular cell adhesion molecule 1
VEGF-A	vascular endothelial growth factor A
Wnt	Wingless-related integration site signalling
